# Quality of Life Outcomes in Vestibular Schwannoma: A Prospective Analysis of Treatment Modalities

**DOI:** 10.1002/lary.32080

**Published:** 2025-02-27

**Authors:** A. Hotchkies, E. Heward, A. Wadeson, C. Heal, S. R. Freeman, S. A. Rutherford, A. T. King, O. Pathmanaban, J. Halliday, G. Whitfield, C. McBain, R. J. Colaco, T. Campbell, S. J. Goh, S. K. W. Lloyd

**Affiliations:** ^1^ Manchester Skull Base Unit, Salford Royal Hospital Manchester UK; ^2^ Faculty of Biology, Medicine and Health University of Manchester Manchester UK; ^3^ The Christie NHS Foundation Trust Manchester UK; ^4^ Manchester University Hospitals NHS Foundation Trust, Manchester Academic Health Science Centre Manchester UK

**Keywords:** conservative management, microsurgery, quality of life, radiosurgery, vestibular schwannoma

## Abstract

**Objective:**

Management options for vestibular schwannoma include microsurgery (MS), stereotactic radiosurgery (SRS), and watch, wait, and rescan (WWR). This study aimed to evaluate changes in patient and disease‐specific quality of life (QoL) outcomes over time, comparing each treatment modality in a matched cohort.

**Methods:**

A prospective cohort study recruited adult patients with sporadic vestibular schwannomas ≤ 3 cm in size undergoing treatment between January 2012 and April 2022 in a single tertiary referral center. Questionnaires were completed at diagnosis and ≥ 12 months posttreatment to assess patient‐reported changes in QoL (Hearing, Dizziness, Tinnitus Handicap Inventories; Penn Acoustic Neuroma QoL questionnaire (PANQOL) and the Short Form‐36 QoL questionnaire (SF‐36)).

**Results:**

In total, 124 patients returned completed questionnaires (MS: 42, SRS: 42, WWR 40). The SRS group had a clinically significant deterioration in their hearing scores posttreatment (*p* = 0.002). Dizziness scores worsened in the MS and WWR groups posttreatment; this did not reach clinical significance. Hearing deterioration was identified in the WWR group over time using the PANQOL domain (*p* = 0.012). The SF‐36 questionnaire showed a significant deterioration in physical functioning, role limitations, and component summary for SRS patients posttreatment (*p* = 0.0018, *p* = 0.0032, *p* = 0.0308). No other significant differences were seen in disease‐specific or general QoL domains when comparing treatment strategies.

**Conclusion:**

Outcomes in similar disease‐specific domains were not consistent across questionnaires. All three treatment modalities appear to result in comparable long‐term disease‐specific QoL outcomes. These findings will enable evidence‐based patient counseling to inform decision‐making.

**Level of Evidence:**

3

## Graphical abstract

One of the first prospective cohort studies looks at quality of life (QOL) outcomes for the three main treatment modalities for vestibular schwannoma. In our study, we found comparable long‐term disease‐specific QOL outcomes, which will allow clinicians to more accurately counsel patients before making life‐changing treatment decisions.

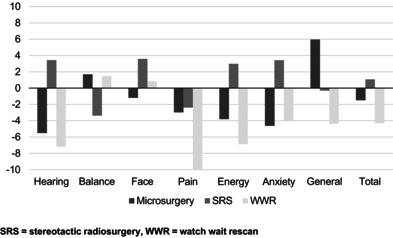

## 1 Introduction

Vestibular schwannomas (VS) are benign tumors of the cerebellopontine angle (CPA) and account for up to 80% of lesions in this region. A recent systematic review suggests that the incidence is between 3 and 5.2/100,000/year with a lifetime prevalence of 1 in 500 [1]. Tumors arise from the Schwann cells surrounding the vestibular nerves. Approximately 7% of patients with VS will have NF2‐related schwannomatosis [2]. Presenting symptoms can include hearing loss, tinnitus, dizziness, facial numbness, and rarely, facial palsy.

Management options include microsurgery (MS), stereotactic radiosurgery (SRS), and watch, wait, and rescan (WWR). The choice of treatment modality is dependent on patient, tumor, and clinician factors, but the default treatment option for most small‐ and medium‐sized tumors is WWR, with only around 30% of tumors showing subsequent growth requiring treatment [3]. Microsurgical outcomes do not start to deteriorate until the tumor reaches around 17 mm in its CPA component, which can help influence microsurgical timing [4]. If untreated, growing tumors become ineligible for SRS because of brainstem compression, and although a minority might be offered fractionated radiotherapy, the majority are treated with surgery.

Whilst historically, key outcome measures following treatment have centered around tumor volume reduction, cranial nerve function, particularly hearing, dizziness, and facial function; in the last 25 years, there has been a greater focus on patient‐reported outcomes, especially quality of life (QoL) [5]. The early literature focused on QoL outcomes of MS, but there has been an increasing number of publications investigating QoL following WWR [6] and SRS, with some attempting to compare modalities. For the last 14 years, there has been a validated disease‐specific QoL instrument, the Penn Acoustic Neuroma Quality of Life (PANQOL) questionnaire, which has replaced generic QoL instruments in assessing QoL in most studies [7, 8, 9, 10].

Despite these advances, there are still considerable deficiencies in the existing literature, with very few studies investigating prospective comparative changes in QoL across all treatment modalities using a disease‐specific instrument [11, 12, 13, 14, 15]. Those that have been published have limitations, including poorly matched patient groups and often lack long‐term follow‐up [12, 16, 17].

This study uses the PANQOL, as well as more generic QoL instruments, to prospectively compare QoL changes between treatment modalities over at least 1 year in small‐ and medium‐sized tumors that are potentially eligible for treatment with any modality.

## 2 Materials and Methods

This prospective cohort study was approved by the East of Scotland research ethics committee (REC 21/ES/0059) and performed at a single tertiary referral center (Salford Royal Hospital, UK).

The primary aim of the study was to compare changes in QoL between WWR, MS, and SRS. The secondary aims were to assess QoL change over time within treatment groups and to investigate factors that might influence QoL.

All adult patients (≥ 18 years) with untreated sporadic VS with a tumor size of 30 mm or less were invited to participate; the latter criteria aimed to try and match the groups as closely as possible. The VS size was calculated as the maximum diameter using axial MRI sequences excluding any intracanalicular component. Patients under 18 years of age and those with NF2‐related schwannomatosis were excluded. Recruitment took place between January 1, 2012 and April 30, 2022.

The initial intention was to only include patients with tumors measuring less than 25 mm. However, to ensure sufficient recruitment to the surgical group, it was necessary to alter the upper size limit to 30 mm. This change was made in 2020. From the study's outset in 2012, patients with tumors greater than 25 mm received the baseline questionnaires. This means a small number of patients (*n* = 5) with tumors between 25 and 30 mm have a longer duration from pre‐ and posttreatment questionnaires compared to those with smaller tumors. This potential bias is mitigated by performing the posttreatment questionnaire ≥ 1 year posttreatment to ensure symptom stability. Of the five patients with tumors ≥ 25 mm, four were in the MS group (MS: 25, 27, 28, and 30 mm, SRS: 25 mm).

Change in PANQOL between treatment groups was the primary outcome used for the sample size calculation. The calculation was based on a one‐way ANOVA with three groups, an effect size of *f* = 0.3, and a power of 0.8. Based on these assumptions, the required sample size was 111 patients. The recruitment number required for each group was therefore determined to be at least 40.

The assessment tools used for this study were the audiovestibular handicap inventories, the PANQOL, and the modified short‐form–36 generic QoL questionnaire. The audiovestibular handicap questionnaires (Hearing Handicap Inventory (HHI), Dizziness Handicap Inventory (DHI), and Tinnitus Handicap Inventory (THI)) consist of 25 symptom and QoL‐based questions. Results are scored from 0 (no handicap) to 100 (maximum handicap). The PANQOL consists of 26 disease‐specific questions divided into 7 domains (hearing, balance, facial, pain, energy, anxiety, and general health) [7]. Patients are scored from 0 to 100, with lower scores indicating a worse disease burden. The 36‐Item Short Form Survey (SF‐36) consists of 36 questions relating to 8 general QoL domains focused on general, physical, and mental well‐being [8]. It also provides summary scores for both physical and mental components. Each domain is scored from 0 (maximum handicap) to 100 (no handicap).

The clinical relevance of any statistically significant changes in audiovestibular handicap, PANQOL, and SF36 scores was assessed using the concept of a minimal clinically important difference (MCID). This is the smallest difference in scores that patients perceive as important and that could lead to a change in management. The MCID for the HHI, DHI, and THI is a 7, 11, and 7‐point change, respectively [18, 19, 20]. The MCID for the PANQOL has recently been determined by Kerezoudis et al. [10]. The median and 25th–75th percentiles for each subdomain are: hearing (13.1, 13–16 points), balance (14.0, 14–19 points), pain (21.0, 20–28 points), face (25.0, 13–36 points), energy (16.0, 15–18 points), anxiety (16.0), and general (13.0). The MCID for the total score is 12.5 (10–15 points). For the SF36, the MCID for component scores has previously been set at 5 points [9].

Patients completed baseline QoL questionnaires at the initial presentation, prior to their initial outpatient appointment. Patients in the WWR group were then sent questionnaires by post at least a year after completion of the initial questionnaires. Those patients in the MS or SRS groups were sent questionnaires by post at least 1 year following the completion of their treatment. If questionnaires were returned incomplete in any way, the patients were contacted by phone to complete the questionnaire, and if that was not possible, the patient was excluded from the study. The eight patients excluded due to incomplete records were distributed over all 3 treatment cohorts (MS group: 2, SRS group: 3, WWR group: 3). A complete dataset was therefore achieved for all included patients.

A pre‐ and posttreatment facial nerve assessment was collected in all patients using the House‐Brackmann scale [21]. The posttreatment facial nerve assessment was performed ≥ 1 year posttreatment to allow for recovery and to align with the posttreatment questionnaire.

The SRS regime used during this study period was 12Gy delivered in one fraction on a Novalis TX linear accelerator.

Results are presented as mean +/− 95% standard deviation (SD) or median +/− interquartile range (IQR). Statistical analysis was performed using StatsDirect and outlined in the supplement (Table S1.). To compare the differences in QoL score change between modalities, a Kruskal–Wallis test was used. To assess changes in QoL within treatment arms, a Wilcoxon Signed Ranks test was used. Due to the multiple tests involved in answering the research questions, a Holm‐Bonferroni sequential correction was also applied. We used linear regression to determine the association between age, sex, and tumor size and the pre‐HHI/THI/DHI; Bonferroni‐corrected *p*‐values for the three tests mean that *p* < 0.0125 was considered significant. We used the Mann–Whitney U test to compare the prescores between sexes, again Bonferroni‐corrected for the three tests. To determine the association between patient and tumor factors and HHI, we used linear regression with post‐HHI as the outcome and pre‐HHI and each of the demographic variables in turn as the independent variables; the regression assumptions were satisfactorily met. However, to determine the demographic characteristics relationship with THI and DHI, we instead had to dichotomize these outcomes as they led to violations of the linear regression assumptions in their raw form and could not be satisfactorily transformed. We then used logistic regression with THI/DHI in their binary forms. To avoid arbitrarily choosing a single cutoff for dichotomizing the THI and DHI, we ran the models with cutoffs at both the median and first quartile and reported both. Holm‐Bonferroni correction was applied to these results. A Mann–Whitney U test was used to compare the posttreatment PANQOL scores and facial nerve function. Significance was defined as *p* < 0.05.

## 3 Results

At this center, during the study period, 1681 patients with VS of any size, were referred; 391 had MS, and 263 had SRS.

**FIGURE 1:**
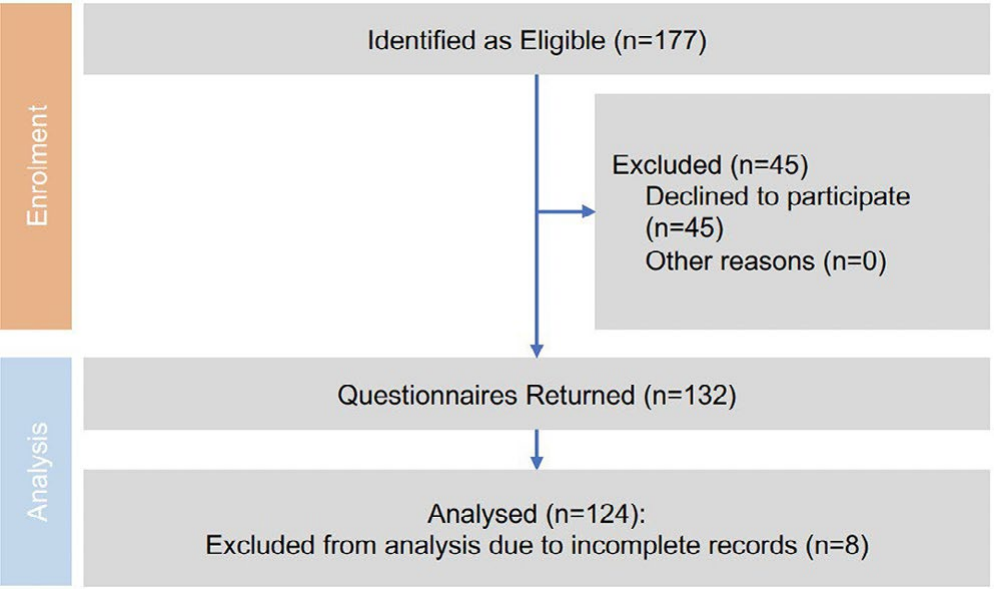
Recruitment data. [Color figure can be viewed in the online issue, which is available at www.laryngoscope.com.]

Of the patients who met the inclusion criteria, 177 patients were identified, 45 patients declined to participate, and eight were excluded due to incomplete records, leaving 124 patients across the three treatment arms (MS = 42; SRS = 42; WWR = 40) (Figure 1). Of the eight patients excluded due to incomplete records, they were distributed over all 3 treatment cohorts. The demographics of each group are summarized in Table 1. The MS group (mean years 53.2, range: 28–74) was significantly younger than the SRS group (mean years 66, range: 47–80, *p* = 0.01) and the WWR group (mean years 70, range: 35–79, *p* = 0.01). Baseline tumor size was larger in the MS group (mean size: 14.3 mm, SD: 6.9) compared to the SRS group (mean size: 10.0 mm, SD: 4.4, *p* = 0.01) and the WWR group (mean size: 8.3 mm, SD: 4.9, *p* = 0.01). The mean duration from baseline to posttreatment questionnaire, across all groups, was 41 months (Table 1).

**TABLE 1:** Patient demographics comparing vestibular schwannoma treatment groups.

Treatment modality	MS (*n* = 42)	SRS (*n* = 42)	WWR (*n* = 40)
Mean age (years) (range)	53.2	66.0	70.0
	(28–74)	(47–80)	(35–79)
Mean tumor size (mm) (SD)	14.3	10.0	8.3
	(6.9)	(4.4)	(4.9)
Mean duration between pre‐ and post‐treatment questionnaire (months) (range)	45	37	42
	(12–102)	(13–71)	(30–52)
Sex (%)			
Male	20 (47.6)	19 (45.2)	21 (52.5)
Female	22 (52.4)	23 (54.8)	19 (47.5)

Abbreviations: MS = microsurgery, SRS = stereotactic radiosurgery, WWR = watch, wait, and rescan.

### 3.1 QoL at Presentation

When comparing the baseline handicap inventory scores between treatment modalities, the MS group had higher HHI scores (median HHI: 39, IQR: 39) compared to the SRS (median HHI: 19, IQR: 33, *p* = 0.045) and WWR (median HHI: 22, IQR: 32, *p* = 0.03) groups (Table 2). Baseline DHI scores were significantly lower in the WWR group (median DHI: 0, IQR: 8) compared to the MS (median DHI: 10, IQR: 34.5, *p* = 0.001) and SRS groups (median score: 10, IQR: 24.5, *p* = 0.004) (Table 2). There was no difference in baseline THI, PANQOL, or SF‐36 scores between treatment modalities (Tables 2, 3 and 4).

**TABLE 2:** Median hearing, dizziness, and tinnitus handicap inventory scores pre‐ and posttreatment comparing vestibular schwannoma treatment groups.

	MS (*n* = 42)			SRS (*n* = 42)			WWR (*n* = 40)		
	Pre	Post	Change	Pre	Post	Change	Pre	Post	Change
HHI	39	41	8	19	31	10**	22	20	0
DHI	10	30	9*	10	14	5	0	6	1***
THI	14	10	0	4	8	0	5	7	0

Abbreviations: DHI = dizziness handicap inventory; HHI = Hearing handicap inventory; MS = microsurgery; SRS = stereotactic radiosurgery; THI = tinnitus handicap inventory.; WWR = watch, wait, and rescan.

* *p* = 0.001.

** *p* = 0.002.

*** *p* = 0.005.

**TABLE 3:** Mean PANQOL questionnaire scores pre‐ and posttreatment by vestibular schwannoma treatment modality.

	MS (*n* = 42)			SRS (*n* = 42)			WWR (*n* = 40)		
	Pre	Post	Change	Pre	Post	Change	Pre	Post	Change
Hearing	46.0	40.5	−5.5	48.4	51.8	3.4	49.5	42.4	−7.2*
Balance	40.4	42.1	1.7	52.4	49.0	−3.4	42.3	43.7	1.5
Face	48.6	47.4	−1.2	50.8	54.4	3.6	42.3	43.1	0.8
Pain	47.0	44.0	−3.0	56.0	53.6	−2.4	46.3	36.3	−10.0
Energy	43.9	40.1	−3.8	51.9	54.9	3.0	46.5	39.6	−6.9
Anxiety	47.6	43.0	−4.6	52.7	56.1	3.4	43.3	39.2	−4.1
General	45.8	51.8	6.0	56.6	56.3	−0.3	47.5	43.1	−4.4
Total	45.6	44.1	−1.5	52.7	53.7	1.1	45.4	41.1	−4.3

Abbreviations: MS = microsurgery; SRS = stereotactic radiosurgery; WWR = watch, wait, and rescan.

* *p* = 0.012.

**TABLE 4:** Mean modified SF‐36 questionnaire scores pre‐ and posttreatment by vestibular schwannoma treatment modality.

	MS (*n* = 42)			SRS (*n* = 42)			WWR (*n* = 40)		
	Pre	Post	Change	Pre	Post	Change	Pre	Post	Change
Physical	48.0	45.9	−2.1	47.1	42.0	−5.1*	50.8	48.7	−2.1
Functioning									
Physical Role	48.3	45.3	−3.0	49.2	42.6	−6.6**	50.8	48.5	−2.3
Limitations									
Bodily pain	40.3	39.1	−1.2	43.0	43.0	0.0	36.9	36.4	−0.5
General health	43.3	42.9	−0.4	46.0	47.1	1.1	45.4	43.0	−2.4
Vitality	44.1	45.8	1.7	52.3	50.2	−2.2	48.8	47.6	−1.2
Social	40.1	41.8	1.7	43.5	42.3	−1.3	41.0	38.7	−2.3
Functioning									
Emotional role	49.2	45.2	−4.0	51.1	45.3	−5.8	51.1	49.9	−1.3
Limitations									
Mental health	44.9	44.2	−0.7	49.1	47.7	−1.5	45.9	46.5	0.6
PCS	45.1	43.6	−1.5	45.7	42.6	−3.1***	46.2	43.6	−2.6
MCS	44.8	44.6	−0.2	50.2	48.3	−2.0	46.8	46.8	−0.1

Abbreviations: MCS = Mental component summary; MS = microsurgery; PCS = Physical component summary; SRS = stereotactic radiosurgery; WWR = watch, wait, and rescan.

* *p* = 0.0018.

** *p* = 0.0032.

*** *p* = 0.0308.

### 3.2 QoL Changes Within Treatment Groups

When assessing change in QoL between baseline and posttreatment time points within treatment groups, there was a significant deterioration in the HHI of patients in the SRS group (*p* = 0.002, *z* = 3.16, median change: 10, IQR: 22.5). Dizziness handicap scores worsened significantly for both the MS (*p* = 0.001, z = 3.19, median change: 9, IQR: 30.5) and WWR (*p* = 0.005, *z* = 2.75, median change 1, IQR: 11.5) cohorts but not in the SRS group (Table 2). Only the SRS group deteriorated enough, within the HHI domain, to reach the MCID. There was no difference seen in the tinnitus inventories.

The PANQOL hearing domain significantly worsened, comparing baseline and follow‐up questionnaires, for the WWR cohort (*p* = 0.012, mean change: 7.2, SD: 18.3) (Table 3). This did not meet the MCID threshold. No other significant changes were seen in other domains in any of the treatment cohorts. The changes seen over time in dizziness handicap were not reflected in the PANQOL dizziness domain. Similarly, the change in PANQOL hearing domain score in the WWR group was not reflected in changes in hearing handicap.

The SF‐36 scores indicate a significant deterioration in physical functioning (*p* = 0.0018, mean change: −5.1, SD: 8.5), physical role limitations (*p* = 0.0032, mean change: −6.6, SD: 10.9), and physical component summary (*p* = 0.0308, mean change: −3.1, SD: 11.7) for the SRS group (Table 4). Changes in physical functioning and role limitation met the MCID. No other significant differences were seen in the other groups.

### 3.3 Differences in QoL Change Between Treatment Groups

**FIGURE 2:**
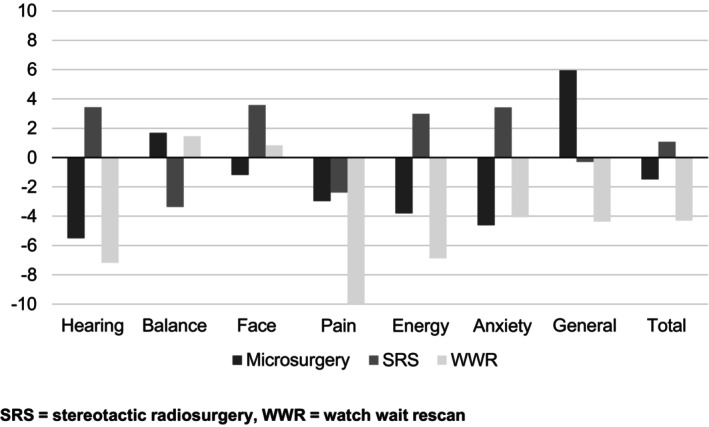
Mean change in PANQOL domain scores between pre‐ and posttreatment by vestibular schwannoma treatment modality.

There were no significant differences in QoL change in any audiovestibular handicap (Table 2). Similarly, there was no significant difference in QoL change in any PANQOL domain (Table 3 and Figure 2).

### 3.4 Factors Influencing QoL Change

Analyzing age, sex, and tumor size, as well as baseline HHI, THI, and DHI, increasing age was significantly associated with worsening THI (Pearsons Corr = −0.24, *p* = 0.009). No other statistical results were demonstrated (Table S1.). After adjusting for baseline HHI, THI, and DHI scores, there was no factor (age, sex, or tumor size) that significantly affected posttreatment scores (Table S1.).

### 3.5 Pre‐ and Posttreatment Facial Nerve Outcomes and Complications

**FIGURE 3:**
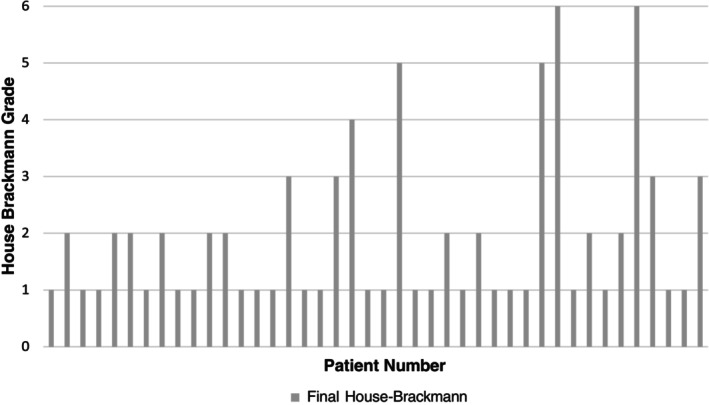
Facial nerve outcomes > 1‐year post‐microsurgery for vestibular schwannoma using the House‐Brackmann scale.

All patients started with normal facial nerve function prior to treatment. In total, 19 MS patients and 2 SRS patients had facial weakness following treatment. Within the MS group, five of these patients were House‐Brackmann Grade 4 or worse (Figure 3). Of those with a postoperative facial nerve weakness, four received facial therapy and one received facial botox. Treatment commenced prior to the posttreatment questionnaire. To our knowledge, no patients underwent facial reanimation surgery. There were no cases of facial palsy in the WWR group.

A worse postoperative House Brackmann score (HB = 1 vs. HB > 1) did not result in a worse facial domain PANQOL score (*p* = 0.929, *z* = 0.089).

Complications were also recorded. Of the surgical patients, there were 3 cerebrospinal fluid (CSF) leaks requiring a lumbar drain. There was one large abdominal hematoma secondary to the fat graft harvest, one pulmonary embolism, and one hospital‐acquired pneumonia. There were no other complications in the SRS or WWR groups.

## 4 Discussion

This study shows that for small‐ and medium‐sized tumors there may be a deterioration in hearing following SRS treatment (median prescore 19 vs. postscore 31, *p* = 0.002) and balance following MS (median prescore 10 vs. postscore 30, *p* = 0.001) or WWR (median prescore 0 vs. postscore 6, *p* = 0.005) based on the HHI and DHI, respectively. There is otherwise no deterioration in QoL at ≥ 1 year following treatment in any of the treatment groups. There was no difference in QoL between the treatment groups.

The early literature, which mainly consists of single modality cross‐sectional assessments using generic questionnaires, generally suggested that QoL was best preserved in those undergoing WWR, with those having MS having worse QoL than those having SRS [6].

Studies comparing modalities are limited in number and are mostly cross‐sectional in design. Kelleher found that, based on the SF36, the QoL of patients undergoing WWR was the same as a control group without VS but that QoL was poorer in social and physical domains in patients having MS [11]. They did not analyze the SRS group because there were only 5 patients in this group. Tos et al. found that there was a greater deterioration in socioeconomic function in patients undergoing surgery compared to those undergoing observation, especially for those with tumors larger than 3 cm [12]. There were no SRS patients included in this study. A fairly recent study of 642 patients with small‐ and medium‐sized tumors published by Carlson et al. used the PANQOL and found that there were better total PANQOL scores and higher facial, balance, and pain PANQOL domain scores in those undergoing observation or SRS compared to those undergoing surgery over a mean follow‐up period of 7.7 years [13]. A similar US national cross‐sectional study including 1362 patients showed that those patients undergoing surgery generally had poorer domain scores for facial function, hearing loss, and pain compared to those undergoing observation or SRS [14]. Other cross‐sectional studies utilizing the PANQOL include those by Robinett et al. [15], McLaughlin et al. [16] and Lodder et al. [22], none of which showed any difference in overall QoL between treatment groups over the long term. McLaughlin et al. did show better hearing domain scores in the WWR group compared to the MS group, and Lodder et al. showed that there was a short‐term detrimental impact on QoL, although this was not broken down by treatment modality [17]. They also showed that facial subdomain scores were better for WWR and SRS than MS and that balance subdomain scores were better for WWR than MS. Fuentealba‐Bassaletti et al. focused on the impact of treatment on vestibular complaints and found that the modality of treatment did not influence dizziness‐related QoL using the PANQOL or DHI [23].

The literature investigating prospective changes in QoL is limited and is summarized in Table 5. The first prospective study was published by Pollock et al. [24]. They compared patients with tumors smaller than 3 cm having MS and SRS using generic QoL instruments (SF36 and handicap inventories) and showed poorer outcomes for physical function and bodily pain and worse dizziness handicap scores for the MS group at 1 year compared to those having SRS who had no decline in any QoL components. A study by Myrseth et al. compared MS with SRS and showed that QoL was better following SRS compared to MS 2 years following treatment using the Glasgow Benefit Inventory (GBI) [25]. General and physical subdomain scores were also worse in the MS group after 2 years. A study by Sandooram et al. showed that, in a relatively small cohort of patients over a 6‐month period, there was no difference in GBI or SF36 scores between those undergoing WWR and those having MS [26]. Di Maio et al. used the SF36 questionnaire and found that there was no long‐term deterioration in QoL in any of the three main treatment modalities and no differences between modalities. As far as the authors are aware, there are only two other prospective studies comparing QoL outcomes between treatment groups using the PANQOL. A study by Carlson et al. demonstrated similar findings to the current study, with no significant difference in total or any subdomain score between the 3 treatment modalities with the exception of an improvement in anxiety scores in the surgery group compared to observation and SRS groups [27] although this did not meet the MCID. This was despite including large tumors and having a relatively short period between baseline and follow‐up assessments. A study by Neve et al. found that all PANQOL scores for WWR, SRS, and MS remained stable at 10 years and none exceeded the MCID [28]. This prospective study is unique because it uses both disease‐specific, including audiovestibular handicap inventories and PANQOL, and general QoL surveys on the same patient population comparing all three treatment modalities.

**TABLE 5:** Prospective studies comparing quality‐of‐life outcomes between treatment modalities for vestibular schwannoma.

Authors	Year	Location	Population	Groups	QoL Instruments
Pollock et al	2006	USA	Adults Sporadic VS	Conservative *n* = 82 Microsurgery *n* = 36 Radiosurgery *n* = 46	DHI, tinnitus survey, headache survey, health status questionnaire
< 3 cm					
Myrseth et al	2009	Norway	Adults Sporadic VS	Radiosurgery *n* = 62 Microsurgery *n* = 28	SF36, Glasgow benefit inventory
< 2.5 cm					
Sandooram et al	2010	UK	Adults Sporadic VS	Conservative *n* = 18 Microsurgery *n* = 15	SF36, Glasgow benefit inventory
≤ 4 cm					
Miller et al	2020	USA	Sporadic VS Unable to ascertain	Conservative *n* = 94 Microsurgery *n* = 16 Radiosurgery *n* = 24	PANQOL
Size or age cutoff/NF2					
Carlson et al	2021	USA	Adults Sporadic VS Any size	Conservative *n* = 78 Microsurgery *n* = 118 Radiosurgery *n* = 48	PANQOL
Neve et al	2022	Netherlands	Adults Sporadic VS Any size	Conservative *n* = 246 Microsurgery *n* = 179 Radiosurgery *n* = 47 Micro and Radio	PANQOL
				Surgery *n* = 15	Decision regret scale

It is interesting to note that universally, across all treatment modalities, the PANQOL scores of patients in the current study were considerably lower than those in both Neve and Carlson's studies for example, the total posttreatment scores for WWR, SRS, and MS were 41.1, 53.7, and 44.1, respectively in this study compared to 74.0, 70.0, and 65.0 in Carlson's paper [27]. The reason for this difference is unclear.

With regards to hearing, patients with sporadic VS tend to have good hearing in their contralateral ear. In addition, many patients will have had effective hearing rehabilitation for their poor hearing ear. These may minimize the impact of unilateral hearing loss to the point where the instruments used are not sensitive enough to identify any handicap. The absence of increased hearing handicap following translabyrinthine surgery in many patients has been demonstrated by other authors [29]. An increase in dizziness handicap was seen in the MS and WWR groups, although there was no change in the balance domain of the PANQOL. The baseline level of dizziness handicap was low in all groups, and increases in handicap were relatively small, probably not enough to be identified by the PANQOL. Dizziness has been shown to be the most important predictor of QoL in patients with VS [6, 22, 23] but as mentioned above, the literature suggests that the modality of treatment does not appear to influence balance outcome [23, 30]. The exception is for patients with severe imbalance who appear to respond positively to surgery [30]. Other authors have shown that tinnitus does not appear to increase over time irrespective of treatment modality [31, 32].

It is also important to bear in mind that some authors have demonstrated a significant detrimental impact on QoL in the first few months following surgery that patients undergoing other treatment modalities do not necessarily experience [24].

There is evidence that facial palsy affects QoL in patients who have had surgery for a VS [17], but the effects on QoL appear to be less profound than one might expect. This may be due to a number of possibilities. The effects of relatively infrequent facial palsy may be averaged out in a large cohort of patients. Mild posttreatment facial weakness, which makes up the majority of patients, may not significantly impact QoL, especially in older patients. Posttreatment facial nerve rehabilitation helps optimize outcomes. The impact on QoL in the presence of severe facial weakness may be offset by the positive effects of having had a large VS removed. It is also possible that the absence of differences between treatment modalities is due to a lack of sensitivity of the currently available assessment tools. Lodder et al. have, however, shown strong content validity for the facial domain of the PANQOL [33]. Carlson et al. have developed another VS‐specific QoL instrument (Vestibular Schwannoma Quality Of Life Index (VSQOL)) and time will tell if this is more sensitive to changes in QoL [34].

The MS group was significantly younger than those having other treatment modalities (mean of 53.2 vs. 66.0 (SRS) and 70.0 (WWR)). This result mirrors the findings of Myrseth et al. Sandooram et al. and Carlson et al. This probably reflects a reluctance to offer SRS to younger patients because of the very small risk of new tumor induction, stroke, and malignant transformation in the long run in those receiving SRS. Older patients may also have additional comorbidities that increase the risk of surgery. The tumor size was significantly larger for MS (mean size of 14.3 mm vs. 10.0 (SRS) and 8.3 (WWR)). Although the study cohort was limited to small‐ and medium‐sized tumors and, as such, the tumors were all eligible for any treatment option, there was still a tendency to offer MS to patients with larger tumors associated with significant brainstem compression. Our results showed no indication that patient or tumor factors affect QoL outcomes in this population of patients with VS ≤ 3 cm in size.

### 4.1 Strengths and Limitations

This study had a number of strengths. It was prospective in its design, adequately powered, used a validated disease‐specific QoL instrument, as well as more generic instruments, compared QoL at baseline in the three main treatment modalities, and followed changes in QoL over at least a year and, in most cases, much longer. It only included small‐ and medium‐sized tumors in adult patients with sporadic VS. Our tertiary referral center covers a wide geographic area comprising patients from varied socioeconomic and ethnic backgrounds. The center receives approximately 150 VS referrals each year. The National Health Service (NHS) removes the financial barrier to accessing imaging and treatment, ensuring equitable access. These factors make this study generalizable.

There were, however, some limitations. First, there was no non‐VS control group. The primary aim of this study was to investigate changes in QoL over time, and although it would be useful to know how the baseline scores differed from a normal population, the authors did not feel a control group was required to answer the research question. Second, there were some significant differences in the demographics and the tumor characteristics between the treatment groups. As outlined in the methods section, the tumor size limit was increased to 30 mm to ensure sufficient recruitment, particularly to the MS group. In this group of 5 patients, the duration between pre‐ and posttreatment questionnaires is longer but is mitigated by collecting all patient questionnaires ≥ 1 year posttreatment.

There are also likely to be other biases in treatment modality selection, such as younger patients with growing tumors tending to select MS over SRS. A further source of selection bias may be present in those returning questionnaires. For example, patients with a smaller disease burden may be less likely to respond to questionnaires, given their VS minimally affects them. We did not perform an analysis to evaluate the skewing effect of asymmetric dropout between groups. Using three questionnaires, while potentially useful, made the completion of the questionnaires more time‐consuming and may also have biased the return rates towards certain segments of the population, e.g., retired patients. The PANQOL, as the only available disease‐specific instrument available at the time of the study, may not be sensitive enough to identify subtle changes in QoL. Similarly, the generic questionnaires may miss subtle aspects of QoL specific to patients with VS.

### 4.2 Conclusion

Whilst there were some statistically significant changes in QoL over time in some domains, they were limited and not consistent across different questionnaires. This study corroborates the findings of two other similarly designed studies in the literature that we failed to find a meaningful difference in QoL between VS treatment modalities, at least based on the instruments used.

## Conflicts of Interest

The authors declare no conflicts of interest.

## Supporting information


**Table S1.** Statistical analysis performed.
