# Supplementary material for: Quality of Life Outcomes in Vestibular Schwannoma: A Prospective Analysis of Treatment Modalities
Source: Laryngoscope. 2025 Feb 27;135(7):2529–37. doi: 10.1002/lary.32080 (PMC12230880; doi:10.1002/lary.32080)
Supplement: Supplementary file 1 — Table S1. Statistical analysis performed. [file LARY-135-2529-s001.docx]

**Supplementary Table S1:** Statistical analysis performed

| **Analysis** | **Outcome** | **Comparison/independent variable(s)** | **Method** |
| --- | --- | --- | --- |
| Comparison of patient demographics at baseline | Age and tumour size | Treatment group A vs B vs C | Unpaired t-test |
| Quality of life at presentation | Baseline handicap inventory scores | Treatment group A vs B vs C | Kruskal-Wallis (non-normality data) |
| Quality of life changes within treatment groups | Handicap score | Pre vs post, within each treatment group | Wilcoxon signed rank |
| Differences in quality-of-life change between treatment groups | Change (pre-post) in handicap score | Treatment group A vs B vs C | Kruskal-Wallis |
| Factors influencing quality of change | Change (pre-post) in handicap score | (in turn): age, sex and tumour size | Simple linear regression, independent variables |
|  |  | as above but adjusting for: HHI, THI, DHI | Multiple linear regression |
| Comparison of post treatment PANQOL facial domain scores and House Brackmann scores | Post treatment PANQOL facial domain score and House Brackmann score (HB=1 verses HB >1) |  | Mann-Whitney U |
